# Rhizobacteria prime the activation of plant defense and nutritional responses to suppress aphid populations on barley over time

**DOI:** 10.1111/nph.70319

**Published:** 2025-06-26

**Authors:** Crispus M. Mbaluto, Sharon E. Zytynska

**Affiliations:** ^1^ Department of Evolution, Ecology, and Behaviour Institute of Infection, Veterinary and Ecological Sciences, Faculty of Health and Life Sciences, University of Liverpool Liverpool UK

**Keywords:** *Acidovorax*, *Bacillus*, differential gene expression, induced responses, microbe‐induced resistance (MIR), plant‐mediated interaction, RNA‐seq transcriptomics

## Abstract

- Interactions between plants and soil microbes are widespread and are documented to modulate plant–insect herbivore interactions. Still, it remains unclear how these shape the overall plant defense responses and the mechanisms in suppressing insect populations.
- We performed bioassays and a time‐series global gene expression analysis of barley (*Hordeum vulgare*) plants to study the underlying molecular pathways induced by two rhizobacteria, *Acidovorax radicis* or *Bacillus subtilis*, against *Sitobion avenae* aphids.
- Root inoculation by *A. radicis* or *B. subtilis* suppressed aphid populations on barley. Analysis of differentially expressed genes and co‐expressed gene modules revealed a combination of rhizobacteria and aphid‐induced plant responses. Aphid feeding triggered distinct plant responses in rhizobacteria‐inoculated barley compared to uninoculated control plants, in phytohormone, glutathione, and phenylpropanoid pathways within 24 h. By day 7, stronger responses were observed in phenylpropanoid and nutrient pathways. By day 21, changes occurred in flavonoid pathways and genes related to aphid‐induced tissue damage and repair.
- Our study suggests that rhizobacteria inoculation of barley against aphids is dynamic and acts through several molecular pathways to modulate and prime plant resistance (defenses) and tolerance (nutrition and growth) to aphids. Future research holds promise for exploiting these interactions for crop protection and pest management in agriculture.

## Introduction

Plants encounter a broad range of insect herbivores that challenge the plant's capability for growth, survival, and yields. In response to insect herbivory, plants activate inducible defenses, including physical and chemical traits, to reduce insect feeding and performance (Mithöfer & Boland, 2012; War *et al*., 2012). Plant‐induced resistance against insect herbivores is regulated by complex interactions among various plant secondary metabolites, phytohormone signaling, nutritional, and structural pathways (Pieterse *et al*., 2009). These pathways interact cooperatively and antagonistically, shaping the specific nature of plant defense responses to the current environment (Pieterse *et al*., 2009; N. Li *et al*., 2019).

Plants naturally associate with a multitude of beneficial soil microbes on roots, often referred to as their second genome (Berendsen *et al*., 2012). These microbes, including fungi and bacteria, provide beneficial functions for the plant, including nutrition provision, growth promotion, and increased resilience to biotic and abiotic stress (Pieterse *et al*., 2009; Goh *et al*., 2013; Shikano *et al*., 2017). When under attack, plants can recruit beneficial bacteria from the surrounding soil to their roots to boost their defenses against herbivores (van Loon *et al*., 1998; Berendsen *et al*., 2012; Pieterse *et al*., 2014). The presence of beneficial microbes before herbivory can provide an added advantage, as rhizobacteria can prime plant defenses that are only triggered upon herbivory (Conrath, 2009; Pineda *et al*., 2010; Kim & Felton, 2013). Primed plants respond more quickly and with stronger defense responses than nonprimed plants (Martinez‐Medina *et al*., 2016). Microbial inoculation of plants can provide access to these beneficial microbes for plants before herbivore arrival, akin to a plant vaccination for stress‐resilience (Berendsen *et al*., 2012). A recent meta‐analysis highlighted the broad potential of rhizobacterial inoculation of plant roots to suppress insects on the plants, with a clear focus on crop pests (Zytynska *et al*., 2024). For piercing and sucking insects such as aphids, significant effects on reducing insect fecundity were dominated by rhizobacteria *Bacillus* strains, while the rhizobacteria *Pseudomonas* spp. and other less studied rhizobacteria showed negative effects on insect fitness, depending on the experimental system (Zytynska *et al*., 2024).

The vast majority of microbe‐mediated induced resistance has primarily been studied using marker genes for plant–microbe–insect studies, focused on a central role of phytohormones. In *Arabidopsis thaliana*, inoculation with *P. fluorescens* primed the plants for enhanced expression of Lipoxygenase 2 (*LOX2*) genes involved in jasmonic acid (JA)‐regulated defense pathways upon aphid *Myzus persicae* feeding (Pineda *et al*., 2012). However, *Bacillus velezensis* inoculation of *A. thaliana* induced higher accumulation of hydrogen peroxide, cell death, and callose deposition in leaves compared to untreated plants, with no role of salicylic acid (SA), JA, ethylene (ET), or abscisic acid in the defense against *M. persicae* aphids (Rashid *et al*., 2017). In tomato, inoculation of *B. subtilis* triggered JA‐dependent as well as JA‐independent responses to reduce whitefly *Bemisia tabaci* (Valenzuela‐Soto *et al*., 2010). In another study, inoculation of *Bacillus* spp. on cotton roots induced gossypol and JA in leaves, leading to reduced feeding by *Spodoptera exigua* larvae (Zebelo *et al*., 2016). In barley, *Acidovorax radicis* was shown to induce plant flavonoids but was not tested in relation to aphid feeding (Han *et al*., 2016), while a later study with aphid feeding again found only limited evidence for the role of SA, JA, or ET, with potential effects again identified for the flavonoid biosynthesis pathway (Sanchez‐Mahecha *et al*., 2022).

Quantifying the complete set of transcripts regulating plant responses to biotic interactions is essential to understanding all key regulatory mechanisms involved in plants' immune responses. With the increased affordability of next‐generation sequencing, RNA‐seq analysis is feasible for large‐scale transcriptomics analyses (Wang *et al*., 2009). While there are many examples of time‐series transcriptomic analysis examining two‐way plant–pathogen, plant–microbe, or plant–insect interactions, there are few studies that examine plant global transcriptomic responses to microbial inoculation and insect feeding combined (but see Harun‐Or‐Rashid *et al*., 2018; Coppola *et al*., 2019; Y. Li *et al*., 2019; Lidoy *et al*., 2025). In rice, systemic resistance against brown planthoppers was induced by rhizobacterium *Bacillus velezensis* YC7010 linked to early defense signaling pathways (leucine‐rich repeat proteins (LRR), JA, ET, and auxin‐related genes), oxidative stress, photosynthesis, and flavonoid biosynthesis, as well as cell wall modification with increased lignin and cellulose (leaf samples collected 48 h post‐planthopper infestation) (Harun‐Or‐Rashid *et al*., 2018). Strong gene expression changes in ET and phenylpropanoid biosynthesis, as well as amino acid metabolism, were observed as a result of the interaction between the fungi *Trichoderma harzianum* and aphids on tomato (samples collected at 48 h post‐aphid infestation) (Coppola *et al*., 2019). Arbuscular mycorrhizal (AM) fungus (*Rhizophagus intraradices*) inoculation of alfalfa increased plant peroxidase and catalase activities, and SA concentration, after 3 d of aphid infestation (Y. Li *et al*., 2019). Finally, a recent study inoculating AM fungus *Funneliformis mosseae* on tomato showed protection against two different chewing herbivores, *Spodoptera exigua* and *Manduca sexta*, via hormonal crosstalk between primed JA‐regulated defenses, fine‐tuned by the ET pathway (samples collected after 24 h insect feeding) (Lidoy *et al*., 2025). While these four studies provide insights into transcriptomic responses within microbe–plant–insect systems, they do not show the dynamic changes that occur over time. This represents an important gap in our understanding of the temporal evolution of microbe‐mediated plant–insect interactions and the molecular mechanisms regulating these interactions.

Here, we present the first comprehensive global transcriptomic study comparing the inoculation of two distantly related rhizobacteria, *Acidovorax radicis* and *Bacillus subtilis*, individually inoculated to barley plant roots, and the effect on a piercing‐sucking aphid herbivore, *Sitobion avenae*, by examining plant gene expression changes in the plant leaves over three time points. *Acidovorax radicis* has been shown to colonize barley roots, leading to a reduction in aphid populations across several experiments (Zytynska *et al*., 2020; Sanchez‐Mahecha *et al*., 2022; Sanchez‐Mahecha *et al*., 2024), driven by reduced survival and reproductive output of the aphids feeding on inoculated plants (Blenkinsopp *et al*., 2024; Xi *et al*., 2024). We previously observed some variation in the expression of PR1, ET‐related, and flavonoid biosynthesis genes in *A. radicis* inoculated barley after 21 d of aphid feeding, but these genes did not explain much of the variation in aphid population sizes (Sanchez‐Mahecha *et al*., 2022). A similar pattern of aphid suppression was observed for *B. subtilis*‐inoculated barley, with effects on aphid reproductive output potentially mediated by aphid density, which may indicate a need to reach a threshold of aphid density (or feeding rate) to trigger defense induction (Blenkinsopp *et al*., 2024). Given that microbe–plant–insect interactions are dynamic, and plant responses to insect herbivory can differ depending on feeding duration and subsequent insect infestation densities, we hypothesized that the impact of the rhizobacteria inoculation and induced transcriptomic changes against the aphid is time‐dependent. We performed a bioassay of barley plants inoculated with rhizobacteria and uninoculated controls, and evaluated aphid fitness by assessing aphid population growth at 7, 14, and 21 d after aphid infestation. We performed transcriptomic (RNA‐seq) analyses to elucidate plant global gene expression using a fully factorial design to disentangle effects of rhizobacteria and aphid feeding; we harvested and sequenced both rhizobacteria inoculated and uninoculated plants after 24 h of aphid infestation (initial aphid feeding), 7 d (aphid colony establishment), and 21 d (sustained aphid population growth) of aphid feeding; no‐aphid controls were harvested at the same time points. Our study sheds light on underlying molecular changes responsible for defense induction and priming of defenses by rhizobacteria with impacts on the aboveground aphid populations.

## Materials and Methods

### Plants, rhizobacteria, and insect–herbivores

We used barley (*Hordeum vulgare* L., var. Irina, obtained from KWS UK Ltd) individually inoculated with two different rhizobacteria species, *Acidovorax radicis* (N35) and *Bacillus subtilis* (B171). The rhizobacteria *A. radicis* was originally isolated from wheat plants (Li *et al*., 2011), and the *B. subtilis* strain was isolated directly from barley plant roots (Schreiner, 2008); both strains were shared with us by colleagues at Helmholtz Zentrum Munich. We used the English grain aphid *Sitobion avenae* (F.) (clonal genotype Fescue maintained as a lab population for > 10 yr) as the aboveground sap‐feeding insect herbivore. All experiments were conducted in a plant growth chamber (Fitotron SGC120, Weiss Technik UK Ltd) set at 18°C, 16 h : 8 h, light : dark photoperiod cycle, and 65% relative humidity.

### Cultivation of rhizobacteria *Acidovorax radicis* and *Bacillus subtilis*

*Acidovorax radicis* was cultivated at 30°C on nutrient agar plates (Difco™ General purpose nutrient broth 8 g l^−1^ + 15 g agar) and grown for 96 h. Cells were harvested and collected into 10 mM MgCl_2_ and homogenized through a 0.8‐μM syringe needle. We resuspended *A. radicis* at a final OD_600_ of 2.0 (estimated 1.6 × 10^9^ CFU ml^−1^). We grew *B. subtilis* for 24 h at 30°C in nutrient‐broth (Difco™ General purpose Nutrient Broth 8 g l^−1^) while shaking at 220 rpm on an Innova 2300 platform shaker (New Brunswick Scientific Europe B.V Nijmegen, the Netherlands). Cells were harvested by centrifuging the broth at 4°C, 4000 ***g*** for 10 min in Sorvall LYNX 4000 Centrifuge (Thermo Scientific, Osterode am Harz, Germany), cleaned in 0.9% NaCl and finally suspended in 10 mM MgCl_2_, and resuspended to a final *B. subtilis* OD_600_ of 2.0 (estimated 1.6×10^9^ CFU ml^−1^). The bacterial suspensions were used immediately for the bioassays.

### Plant growth condition, rhizobacteria inoculation, and aphid infestation

We used a fully factorial experimental design including three inoculation treatments (control, *A. radicis*, *B. subtilis*) and two aphid treatments (with aphids or without aphids), with six replicates. Before germination, barley seeds were surface‐sterilized by immersion in 4% sodium hypochlorite (bleach) solution for 1 min, and then rinsed thoroughly under running tap water. We performed this mild surface sterilization as a precautionary step to ensure a controlled starting condition for our experiments and reduce the likelihood of interference from external contaminants and microbes. For rhizobacteria inoculation, seeds were soaked in the suspension of *A. radicis* or *B. subtilis* for 2 h, whereas control seeds were mock inoculated by soaking in 10 mM MgCl_2_ for 2 h (following Zytynska *et al*., 2020; Sanchez‐Mahecha *et al*., 2022). The inoculated seeds were then germinated in seed‐trays in nonsterilized potting substrate (Levington's Advance F1 Compost, low nutrient); nonsterilized potting substrate was used since we do not aim to understand these interactions under sterile conditions, as the crop inoculation effect must be transferable to the field. When seedlings were 6 d old (counting from sowing day), we measured the root and shoot lengths to select homogeneous plants for the bioassays. We transplanted the seedlings into individual pots (9 cm diameter × 8.5 cm height) in individual water containers and immediately watered, each with 60 ml of tap water, and afterward as required (*c*. every 3 d). The seedlings were grown in the plant growth chamber for 24 h until the shoots were *c*. 9 cm tall before being used for the experiment. Aphid infestation was achieved by placing 6 *S. avenae* apterous (unwinged) 4^th^ instar aphids at the base of the plant using a fine paintbrush, except for the 21‐d treatment, where we only introduced two aphids to avoid reaching such high aphid population sizes that could lead to plant death. Every plant pot was then covered with a fine white net, using a frame, to eliminate aphid movement across plants. Further, we placed the plant pots onto individual saucers to avoid soil or rhizobacteria cross‐contamination. At each harvesting time point of 24 h, 7 d, and 21 d post‐aphid infestation, we first counted the number of aphid adults and offspring to estimate the level of herbivory, and then recorded plant root and shoot lengths. For 21 d experiment plants, we counted the number of aphids at 7, 14, and 21 d post‐aphid infestation to evaluate aphid population growth over time. In all experiments, at harvest time points, aphids were carefully removed from plants by brushing with a fine‐hair paintbrush, which stimulated them to remove their feeding stylet and drop off the plant. Aphid‐infested shoot tissues, including the whole shoot from the 24 h, and top young leaves where the majority of aphid feeding occurs from the 7‐ and 21‐d experiments, were collected and flash‐frozen in liquid nitrogen and stored at −80°C. Control plant tissues were harvested using the same approach as described for the aphid‐infested plants. Sample harvesting for each time point was conducted in the morning (9–10 AM for the 24 h and 7 d, and 9 AM−2 PM for the 21 d) to standardize the time point in relation to the circadian clock.

### RNA extraction and sequencing

We extracted total RNA from leaf samples collected at the three study time points (24 h, 7 d, 21 d). We used 50 ± 5 mg (fresh weight) of ground leaf material and extracted the RNA using the TRIzol method (Thermo Fisher Scientific, UK), according to the manufacturer's instructions. All samples were treated with 2 U μl^−1^ of RNAse‐free DNase I (Thermo Fisher Scientific) to remove any traces of DNA. The RNA quantity and quality were confirmed using the ND‐1000 UV/vis spectrophotometer (NanoDrop Technologies, Wilmington, DE, USA), and by gel electrophoresis (1% molecular grade agarose). A Qubit 4 Fluorometer (Life Technologies Holdings Pte Ltd, Woodland HDB Town, Singapore) was used to assess the RNA integrity. Samples whose RNA integrity quality score was ≥ 7 were selected for library preparation and sequencing at the Centre for Genomic Research, University of Liverpool, UK. We sequenced four replicates from every treatment combination (72 samples). The preparation of RNA libraries involved the depletion of ribosomal RNA via poly‐A selection using the NEBnext Poly(A)mRNA magnetic isolation module. The poly A‐enriched samples were then used as input in the NEBnext Ultra II RNA kit (NEB, USA) and processed following the manufacturer's instructions. Libraries were validated and quantified using the Illumina library quantification kit (KAPA, Library Quant Kit (Illumina) qPCR master mix 2×) on the Roche Light Cycler LC480II (Roche Diagnostic Ltd, Rotkreuz, Switzerland) following the manufacturer's recommendations. Libraries were sequenced on an Illumina NovaSeq 6000 platform (Illumina, San Diego, CA, USA) following standard workflow over one lane of an S4 flow cell. The sequencing produced 2 × 150 bp paired‐end raw reads with a mean sequencing depth of *c*. 40 million reads per sample.

### Transcriptomic analysis

The raw Illumina reads were first pre‐processed for initial quality assessment using the in‐house pipeline at the CGR‐genomics, University of Liverpool, UK. Base calling and de‐multiplexing of indexed reads were carried out using CASAVA v.1.8.2 conversion software (Illumina) to produce read sequences in fastq format. Clean reads were obtained by using Cutadapt v.1.2.1 software by trimming the Illumina adapter sequences on the raw fastq files (Martin, 2011). We removed low‐quality bases using Sickle v.1.200 software set at a minimum window quality score of 20. Raw reads shorter than 20 bp were removed from the dataset. The clean high‐quality paired‐end raw reads were pseudo‐aligned to the barley reference transcriptome sequence (Ensembl Plants; Hordeum vulgare.MorexV3_pseudomolecules_assembly.cdna.all.fa) using Kallisto v.0.46.2 (Bray *et al*., 2016). The number of raw reads mapped to each gene was counted using the kallisto quant function.

### Differential gene expression and functional enrichment analyses

We used a statistical computing environment R v.4.3.2, in RStudio v.2022.12.0 + 353 and Bioconductor v.3.16 for differential gene expression and functional enrichment (Gentleman *et al*., 2004; Huber *et al*., 2015; R Core Team, 2024; RStudio Team, 2024). First, we summarized the reads (transcripts) count data at the gene level using the tximport R package (Soneson *et al*., 2015). Then we normalized the data using the trimmed mean of M values (TMM) method in edgeR (Robinson *et al*., 2010). We filtered genes with < 1 count per million (CPM) in *n* + 1 (where *n* is the number of biological replicates in the treatment group). The single and interactive effects of rhizobacteria and aphids on gene expression were determined using PERMANOVA models in R, including rhizobacteria treatment (control, *A. radicis*, or *B. subtilis*), aphids (*S. avenae*), and their interaction as explanatory variables. Then, we used the voom function in the limma R package for variance stabilization (Ritchie *et al*., 2015). The limma R package and decideTests function were used to select differentially expressed genes at FDR < 0.05, absolute logFC ≥ +2 or ≤ −2 for each study time point (Benjamini & Hochberg, 1995). Gene ontology (GO) enrichment analyses were performed using a self‐curated annotation file based on the *Hordeum vulgare* gene (MorexV3_pseudomolecules_assembly) dataset in the Ensembl Plants public database. We extracted the TERM2NAME and TERM2GENE mapping from the GO.db R package (Carlson, 2022) and used it for GO enrichment over‐representation analysis. Significantly enriched GO categories, including biological process (BP), molecular functions (MF), or cellular components (CC) terms, were determined at *P*‐value cutoff ≤ 0.05, *P*‐value adjusted by the Benjamini–Hochberg method, and *q*‐value cutoff ≤ 0.2. Kyoto Encyclopedia of Genes and Genomes (KEGG) enrichment analyses were achieved by converting the differentially expressed genes into rice orthologs (*Oryza sativa* Japonica group) using the web application g:Profiler2 (Kolberg *et al*., 2020). This approach was ideal because barley is not yet fully annotated and lacks enrichment KEGG datasets. Significantly enriched KEGG pathways were determined at *P*‐value cutoff ≤ 0.2, *P*‐values were adjusted by the Benjamini–Hochberg method, and *q*‐value cutoff ≤ 0.2 (Li, 2022).

### Gene co‐expression analyses

We performed weighted gene co‐expression network analyses (WGCNA) using the Wgcna R package to detect co‐expressed gene modules related to the impact of rhizobacteria against aphids (Langfelder & Horvath, 2008). We used raw read count data and filtered out genes with less than 50 counts across all treatments per study time point. Then we normalized the data and calculated the power value for gene correlation using the pickSoftThreshold function (Supporting Information Fig. S1). We used the block‐wise modules function to construct block‐wise networks with the following parameters: power = selected power from fitIndices, networkType = signed, maxBlockSize = 5000, TOMType = signed, minModuleSize = 30, mergeCutHeight = 0.25, and plotted dendrograms of the identified gene modules (Fig. S1). Next, we assessed the module–trait relationship by performing linear models in R (limma) to calculate eigengene (hypothetical central genes) for each module, and extracted the gene module exhibiting the largest significant difference across treatment groups. Further, we extracted genes in the selected modules of interest and performed functional enrichment in the KEGG database as described above for differentially expressed genes. Gene connectivity based on edge weights (ranging from 0 to 1) was determined by the topological overlap matrix. The weight across all edges of a node was summed and used to define the level of connectivity. Nodes with high connectivity (≥ 100) were presented in the network. Module–membership or intramodular connectivity measure (connectivity of a gene to all other genes) within the same module was used to define hub genes (driver genes) in each module per time point; module eigengene (ME)‐based connectivity (kME) values ≥ 0.8 were considered as the hub genes for the particular module. Next, we identified the hub genes that had high connectivity (≥ 100) within the network as the main hub gene of interest or drivers of the whole module. Similarly, we performed functional enrichment of the main hub genes of interest in the KEGG database as described above.

## Results

### Rhizobacteria suppress aphid populations on barley

**Fig. 1:**
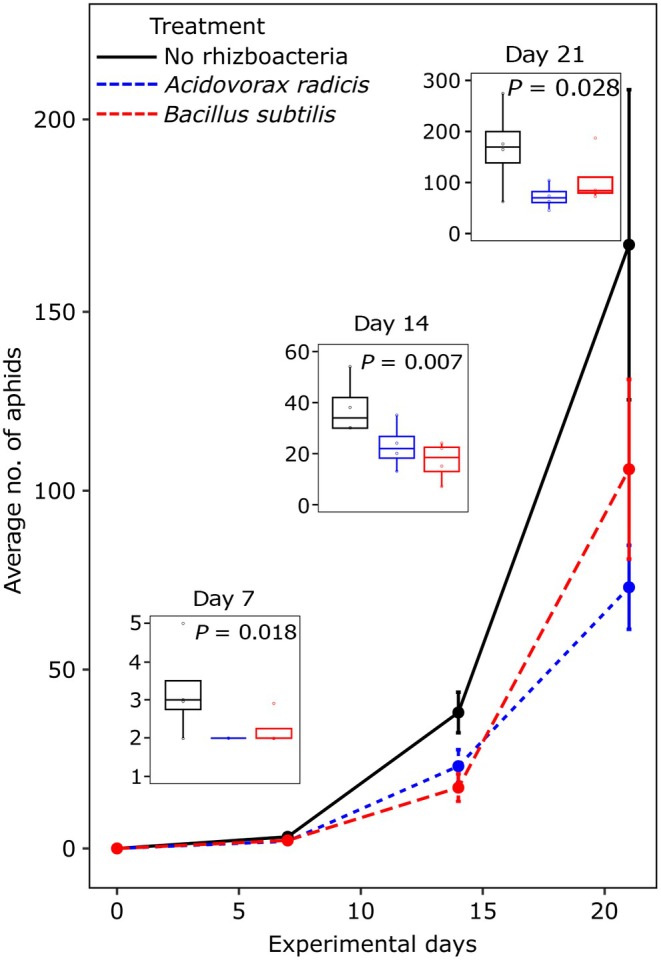
Impact of barley plant inoculation with rhizobacteria *Acidovorax radicis* (blue) or *Bacillus subtilis* (red) and control‐inoculated plants (black) on the population growth of the aphid *Sitobion avenae*. In the main panel, the line plot shows data across the experimental time. Error bars are mean ± SE (*n* = 4, representing the four plant replicates sequenced in this study for day 21). Inset boxplots show data within each time point with associated statistical significance (tested using standard linear models per time point). Boxplots show the median (horizontal line), interquartile range (box), and data range excluding outliers (whiskers). Points represent individual data values.

We first examined the effect of rhizobacteria inoculation on the performance of the aphid. Overall, our results demonstrated significant suppression of the aphid *S. avenae* population on plants inoculated with either rhizobacteria (*A. radicis* or *B. subtilis*) compared to control plants, and across the three time points (7 d: *F*_2,11_ = 8.47, *P* = 0.018; 14 d: *F*_2,11_ = 12.98, *P* = 0.007; and 21 d: *F*_2,11_ = 6.93, *P* = 0.028; Fig. 1). Rhizobacteria inoculation significantly increased early shoot growth (day 1: *F*_2,20_ = 9.51, *P* = 0.001, Fig. S1) but this effect was lost at later time points (day 7: *F*_2,20_ = 1.58, *P* = 0.231; day 21: *F*_2,19_ = 0.37, *P* = 0.698). Aphid infestation significantly reduced plant shoot growth by day 21 (*F*_1,19_ = 9.39, *P* = 0.006; Fig. S2). There were no significant effects of rhizobacteria or aphids on root growth across time points (Fig. S3).

### Rhizobacteria and aphids trigger distinct transcriptome profiles in barley

To unravel the plant responses altered by rhizobacteria resulting in suppression of the aphid populations, we performed RNA‐seq analyses at three time points, including 24 h (initial aphid feeding), 7 d (aphid colony establishment), and 21 d (sustained aphid population growth) after aphid infestation. We constructed 72 RNA libraries and obtained an average of 38 M reads per library. We mapped 74% of the clean raw reads to the barley reference transcriptome (Table S1) and identified *c*. 24 000 annotated genes.

**Fig. 2:**
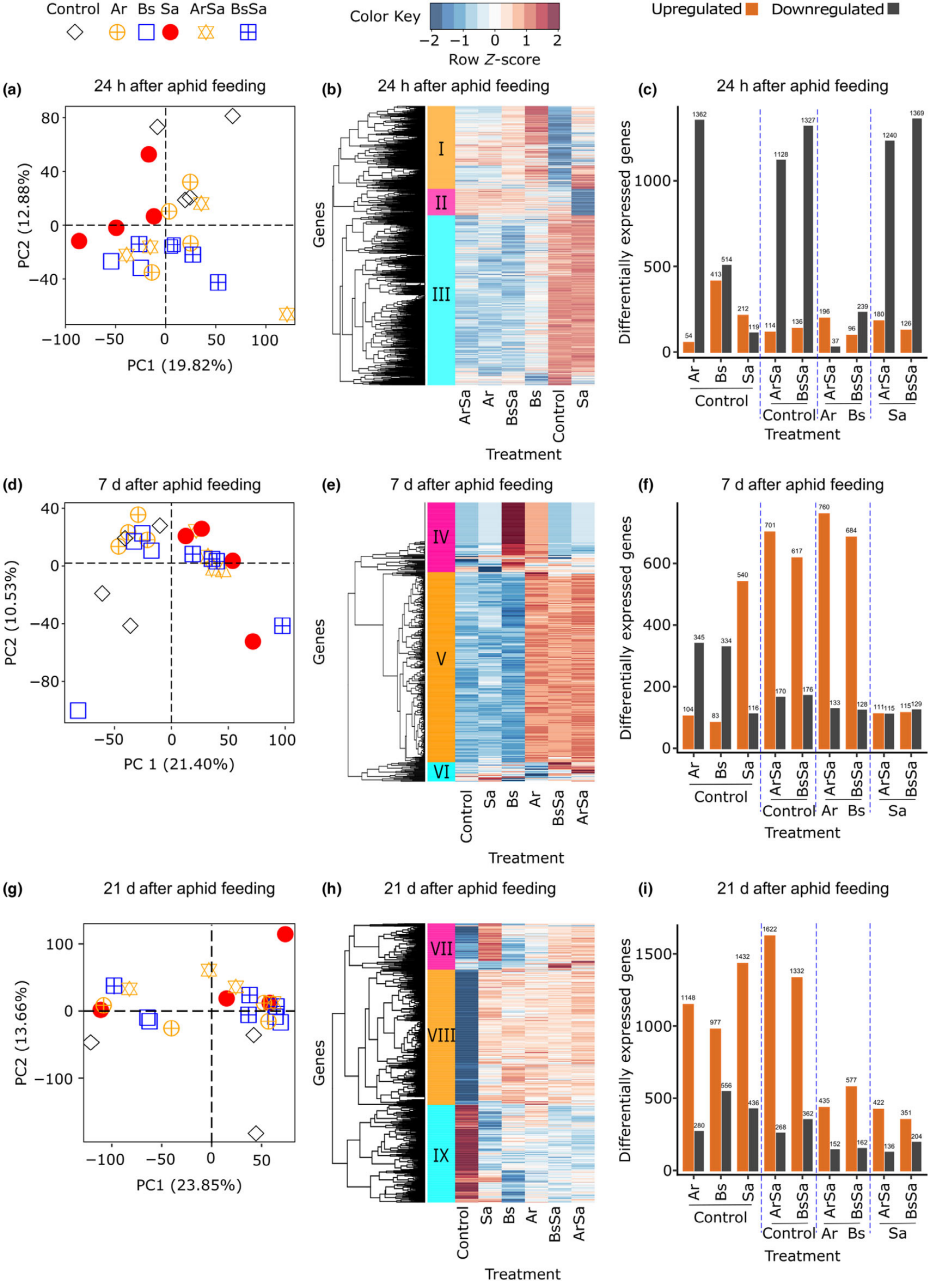
Induced transcriptome profiles of barley leaves upon rhizobacteria root colonization and shoot herbivory. Overview of the global transcriptome profile and differentially expressed (DE) genes in leaves of barley plants without treatment (*Control*), inoculated with rhizobacteria *Acidovorax radicis* (Ar) or *Bacillus subtilis* (Bs), infested with aphid *Sitobion avenae* (Sa), or inoculated with rhizobacteria and infested with aphids (ArSa or BsSa). The panels are arranged per time point based on aphid feeding duration, including 24 h in (a–c), 7 d in (d–f), and 21 d in (g–i). (a, d, g) In the first column are principal component analysis showing scores (treatment groups); (b, e, h) in the middle column are heatmaps displaying relative gene expression for genes identified as differentially expressed in at least one comparison (each line or row represents a unique gene, and each column represents a unique treatment group). The expression direction is defined by colors; that is, blue indicates downregulation, white indicates no change, while red indicates upregulation. (c, f, i) In the third column are bar plots showing the number of DE genes per treatment relative to their respective controls, arranged per comparison with the ‘control’ defined below the horizontal line.

We found that the modulation of the barley transcriptome by rhizobacteria or aphids differed significantly across the time points (PERMANOVA: *F*_2,63_ = 31.15, *R* ^2^ = 0.46, *P* < 0.001). We detected significant main effects of aphid feeding (averaged across time points: *F*_1,63_ = 4.35, *R* ^2^ = 0.03, *P* = 0.002) and rhizobacteria root inoculation dependent on time (interaction between bacteria and time point: *F*_2,63_ = 2.25, *R* ^2^ = 0.03, *P* = 0.012). Because our experiment involved two different bacterial species, we examined for species‐specific patterns. However, we found that rhizobacteria‐induced changes in the barley global transcriptomic profile were not specific to the bacterial strain (main effect of strain identity: *F*_1,40_ = 0.63, *R* ^2^ = 0.01, *P* = 0.711). Our results suggest that the observed changes in the barley transcriptome are explained by interactive effects across our treatments (Fig. 2). At 24 h post‐aphid feeding, we observed strong, significant interaction effects (aphid‐by‐rhizobacteria presence) on transcriptomic changes (Fig. 2a; Table 1). After 7 and 21 d of aphid feeding, the most significant factor modulating plant transcriptomic changes was the aphids, with decreasing strength of effects of rhizobacteria inoculation (Fig. 2d,g; Table 1).

**Table 1:** Permutation multivariate analyses of variance (PERMANOVA) results of transcriptome expression upon rhizobacteria root colonization and shoot herbivory.

Source of variation	Time points											
	24 h				7 d				21 d			
	df	*F*	*R* ^2^	*P*	df	*F*	*R* ^2^	*P*	df	*F*	*R* ^2^	*P*
Rhizobact.P/A	1, 19	**4.17**	**0.16**	**< 0.001**	1, 20	1.47	0.06	0.074	1, 19	1.42	0.06	0.104
Aph	1, 19	1.16	0.04	0.245	1, 20	**4.48**	**0.17**	**< 0.001**	1, 19	**2.03**	**0.09**	**0.014**
Rhizobact.P/A*Aph	1, 19	**2.19**	**0.08**	**0.007**	1, 20	0.83	0.03	0.693	1, 19	1.23	0.05	0.187

*P*‐values in bold indicate statistical significance with *P* < 0.05. Aphid: *Sitobion avenae*; Plant: barley *Hordeum vulgare*; Rhizobacteria: *Acidovorax radicis* and *Bacillus subtilis*. Aph, aphid; df, degrees of freedom; Rhizobact. P/A, rhizobacteria presence/absence.

Next, we obtained differentially expressed genes and performed hierarchical clustering, revealing three main gene clusters varying across the treatments and time points (Fig. 2b,e,h). After 24 h of aphid feeding, the most upregulated genes were contained in Cluster III from control and aphid‐infested plants, while this cluster was predominantly downregulated in plants inoculated with rhizobacteria regardless of aphid presence (Fig. 2b). After 7 d of sustained aphid feeding, Cluster V contained the majority of differentially expressed genes. These genes were highly expressed in *A. radicis*‐inoculated plants with and without aphid infestation, and in *B. subtilis*‐inoculated plants only when aphids were present (Fig. 2e). After 21 d of aphid feeding and population growth, there was a notable separation of control plants from all other treatments, with less variation in gene expression among all inoculated plants than in the other time points (Fig. 2h). To further examine the interactive effects, we compared the different treatments relative to their respective controls. Gene expression increased over time, with the combined (interactive) effect of rhizobacteria inoculation and aphid infestation driving the majority of gene expression differences (Fig. 2c,f,i; Table S2). At day 7 when aphid colonies were establishing, we observed a strong induction of aphid‐related genes (Fig. 2f *Sa* vs *control*, *ArSa* vs *Ar*, and *BsSa* vs *Bs*; Table S2).

**Fig. 3:**
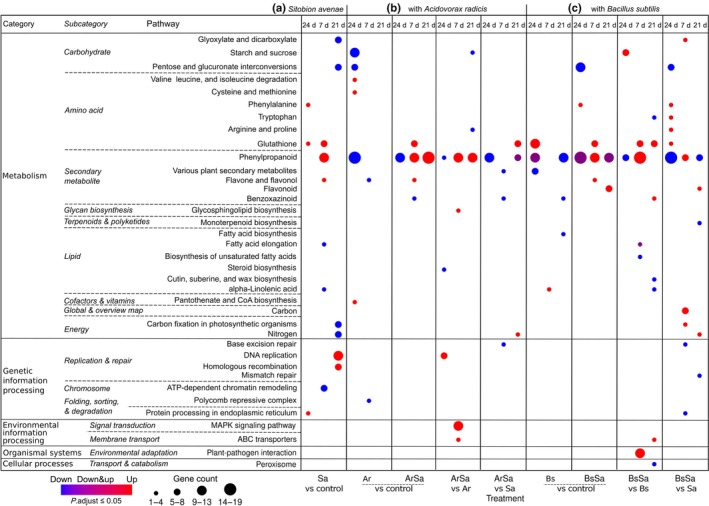
Kyoto Encyclopedia of Genes and Genomes (KEGG) pathway enrichment analysis of differentially expressed genes in barley leaves upon rhizobacteria root colonization and aphid herbivory. This bubble plot shows the enriched KEGG pathways of upregulated and downregulated differentially expressed genes in leaves of barley without treatment (*Control*), infested with the aphid *Sitobion avenae* (*Sa*), inoculated with the rhizobacteria *Acidovorax radicis* (*Ar*) or *Bacillus subtilis* (*Bs*), or inoculated with rhizobacteria and infested with aphids (*ArSa* or *BsSa*). In *Sa*, *ArSa*, and *BsSa* plants, the aphid *S. avenae* was allowed to feed on plants for 24 h, 7 d, and 21 d. (a) Genes induced by *Sa* relative to *Control*. (b) Treatment groups inoculated with *Ar*: *Ar* vs *Control* are genes altered by *Ar* in the absence of aphids. *ArSa* vs *Control* are genes induced by both rhizobacteria and aphids relative to *Control*. *ArSa* vs *Ar* shows the aphid (*Sa*)‐induced genes in rhizobacteria (*Ar*)‐inoculated plants. *ArSa* vs *Sa* shows the rhizobacteria (*Ar*)‐induced genes in aphid (*Sa*)‐infested plants. (c) Treatment groups inoculated with *Bs*: *Bs* vs *Control* are genes altered by *Bs* in the absence of aphids. *BsSa* vs *Control* are genes induced by both rhizobacteria (*Bs*) and aphids (*Sa*) relative to *Control*. *BsSa* vs *Bs* shows the aphid (*Sa*)‐induced genes in rhizobacteria (*Bs*)‐inoculated plants. *BsSa* vs *Sa* shows the rhizobacteria (*Bs*)‐induced genes in aphid (*Sa*)‐infested plants.

Using KEGG pathway enrichment analyses, we showed that the differentially expressed genes were enriched in 16 KEGG subcategories belonging to five main categories, namely, metabolism, genetic information processing, environmental information processing, organismal systems, and cellular process (Fig. 3). Notably, our transcriptome data showed that metabolism was most affected, especially the glutathione (amino acid) and phenylpropanoid (secondary metabolite) pathways. The main modulated glutathione gene was *glutathione‐S‐transferase* (GST), while several phenylpropanoid genes were altered, including *phenylalanine‐ammonia lyase* (PAL), *Caffeic acid‐O‐methyltransferase* (CaOMT), *Cinnamoyl‐CoA reductase* (CCR), *Flavonoid 3′‐O‐methyltransferase*, and *Sinaply‐alcohol dehydrogenase* (SAD). In addition to these phenylpropanoid genes, we found modulation of *peroxidases*, highlighting their involvement in regulating the phenylpropanoid pathway (Tables S3– S6).

Overall, aphid infestation of plants without rhizobacteria inoculation upregulated genes in the glutathione pathway at 24 h and 7 d, and the phenylpropanoid pathway after 7 d of feeding (Fig. 3, *Sa* vs *control*). In rhizobacteria‐inoculated plants without aphid infestation, *B. subtilis* increased glutathione at the 24 h time point (Fig. 3, *Bs* vs *control*), while there were genes both up‐ and down‐regulated in the phenylpropanoid pathway, indicating dynamic expression in this pathway (Fig. 3, *Bs* vs *control*). For *A. radicis*‐inoculated plants (no aphids), there was strong suppression of the phenylpropanoid pathway at the 24 h time point, with no induction of the glutathione pathway (Fig. 3, *Ar* vs *control*). With aphid feeding, we still observed *A. radicis* suppression of the phenylpropanoid pathway at the 24 h time point with no suppression at day 7 but further suppression effects at 21 d (Fig. 3, *ArSa* vs *Sa*). Aphid induction of phenylpropanoid defenses continued through to 21 d on inoculated plants (Fig. 3, *ArSa* vs *Ar*), which was not observed on uninoculated plants (Fig. 3, *Sa* vs *Control*). A similar pattern was observed for *B. subtilis* plants, where the rhizobacteria suppressed the phenylpropanoid pathway at the 24 h and 21 d time points, but increased expression at day 7 (Fig. 3, *BsSa* vs *Sa*), potentially mitigating some of the aphid induction of these defenses (Fig. 3, *BsSa* vs *Bs*).

A number of other pathways across the main categories showed variable regulation across our treatments. After 21 d of aphid feeding in the absence of rhizobacteria, aphids downregulated carbohydrates, including *glyoxylate* and *dicarboxylate* and *pentose* and *glucuronate* interconversion. Starch and sucrose carbohydrate pathways, such as *beta 1–4 glucanase* and *α‐glucoside*, were downregulated by *A. radicis* inoculation at the early time point (Fig. 3, *Ar* vs *Control*), while *B. subtilis* inoculation only suppressed these upon aphid feeding (Fig. 3, *BsSa* vs *Sa*). *Bacillus subtilis* further induced expression of genes for carbohydrate‐related *glyoxylate* and *dicarboxylate*, including *ribulose 1,5‐bisphosphate carboxylase* at day 7 (Fig. 3, *BsSa* vs *Sa*), which were suppressed by aphids on control plants in the later time point (Fig. 3 *Sa* vs *control*). We also found upregulation of *replication* and *repair genes* at day 21 that may reflect plant responses to increased aphid population sizes and subsequent damage (Fig. 3, *Sa* vs *control*).

### Gene co‐expression reveals rhizobacteria prime defense and nutritional responses in barley to suppress aphids

**Fig. 4:**
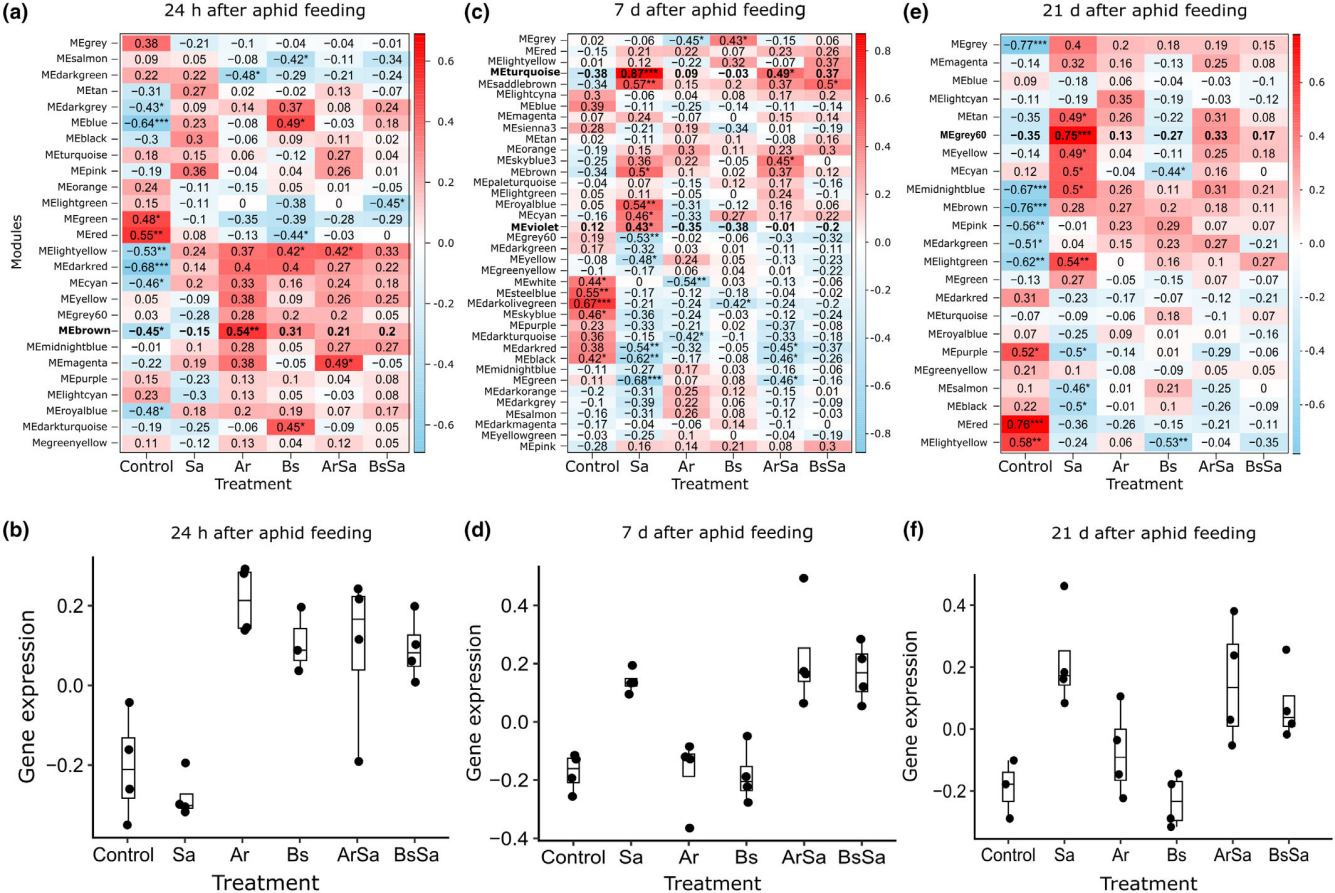
Overview of gene co‐expression and patterns of selected modules of interest over time points in barley leaves upon rhizobacteria root colonization and shoot herbivory. (a, c, e) Heatmaps showing the relationship between each gene co‐expression module and the treatment groups. The treatment groups include barley plants without treatment (*Control*), infested with the aphid *Sitobion avenae* (*Sa*), inoculated with the rhizobacteria *Acidovorax radicis* (*Ar*) or *Bacillus subtilis* (*Bs*), or inoculated with rhizobacteria and infested with aphid (*ArSa* or *BsSa*). In *Sa*, *ArSa*, and *BsSa* plants, the aphid *S. avenae* was allowed to feed on plants for 24 h (a), 7 d (c), and 21 d (e). Each row represents a module eigengene (ME), which is the dominant expression profile of genes in the module, and is named randomly by color. Each column represents a treatment group. The color of each cell represents the association coefficient between ME and the treatment group; red denotes a positive relationship, while blue indicates a negative relationship between the module and treatment. Asterisks indicate a significant correlation. (b, d, f) Are the selected modules of interest per time point. (b) Brown module at 24 h, (d) combined turquoise and violet modules at 7 d, and (f) gray60 module at 21 d post aphid infestation. The treatment groups are labeled as above. Boxplots show the median (horizontal line), interquartile range (box), and interquartile range (whiskers). Points represent individual data values.

To identify groups of genes with similar functions or involved in same pathway that respond to rhizobacteria inoculation and/or aphid feeding, we performed WGCNA. We identified 26, 38, and 23 gene modules across the three time points, containing between 31 and 9750 genes (Table S7). The co‐expressed modules were randomly color‐coded, and we present four that showed large differences across the treatments and time points (Fig. 4a,c,e; Table S8). After 24 h of aphid feeding, genes in the module brown (Fig. 4a) were upregulated in plants inoculated with rhizobacteria with and without aphid feeding, and downregulated in uninoculated plants with and without aphid (MEbrown *n* = 2555, *F* = 4.61, *P* = 0.018; Fig. 4b; Table S8a). After 7 d of aphid feeding, genes in the modules turquoise and violet (Fig. 4c) were upregulated in all plants infested with aphids regardless of rhizobacteria inoculation (MEturquoise *n* = 3353, *F* = 4.03, *P* = 0.022, and MEviolet *n* = 90, *F* = 4.12, *P* = 0.022; Fig. 4d; Table S8b). After 21 d of aphid feeding, genes in the module gray60 (Fig. 4e) were upregulated across the aphid‐infested plants, while *A. radicis*‐inoculated plants without aphids showed intermediate expression (MEgray60 *n* = 85, *F* = 3.40, *P* = 0.059; Fig. 4f; Table S8c).

**Fig. 5:**
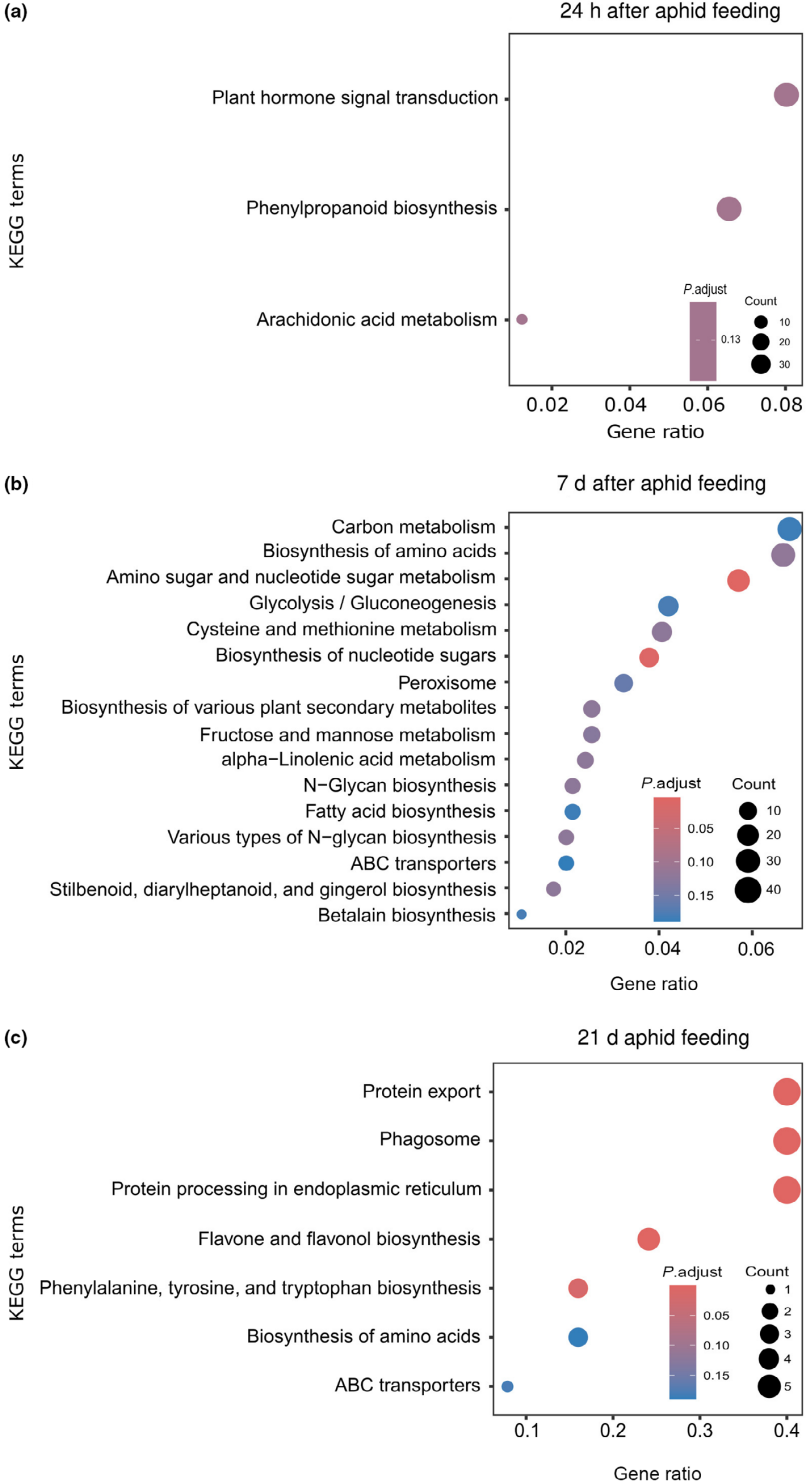
Kyoto Encyclopedia of Genes and Genomes (KEGG) pathway enrichment analysis of co‐expressed gene module in barley leaves upon rhizobacteria root colonization and shoot herbivory. Panels represent the enriched KEGG pathways of the genes in each selected co‐expressed module across all treatment groups, presented as bubble plots. The treatment groups were barley plants without treatment (*Control*), infested with the aphid *Sitobion avenae* (*Sa*), inoculated with the rhizobacteria *Acidovorax radicis* (*Ar*) or *Bacillus subtilis* (*Bs*), or inoculated with rhizobacteria and infested with the aphid (*ArSa* or *BsSa*). In *Sa*, *ArSa*, and *BsSa* plants, the aphid *S. avenae* was allowed to feed on plants for (a) 24 h, (b) 7 d, and (c) 21 d. Gene ratio (*x*‐axis) is the proportion of the study genes mapping to the genes annotated for the specific KEGG pathway. KEGG terms (*y*‐axis) are the pathways associated with the study genes.

Next, we performed KEGG enrichment analyses of the genes in the selected modules of interest at each time point. After 24 h of aphid feeding, we found co‐expressed genes (‘brown’ module) enriched for plant hormone signal transduction, phenylpropanoid biosynthesis, and arachidonic acid metabolism (Fig. 5a). In the plant‐hormone signal transduction, we found the co‐expressed genes included *ubiquitin ligase complex*, *jasmonyl‐L‐isoleucine synthase* for JA biosynthesis, *ET receptor* and *response* for ET signaling, *auxin responsive protein, auxin transporter*, *indole‐3‐acetic acid protein* for AUX responses, *histidine phosphotransfer protein* for CK signaling, and *mitogen‐activated protein kinase kinase 4* for activation of the mitogen‐activated protein kinase (MAPK) (Tables 2, S9a). For the phenylpropanoid pathway, we found seven main genes, including *phenylalanine–ammonia lyase* (PAL), *4‐coumarate‐CoA*, *cinnamyl alcohol dehydrogenase* (CAD), *sinapyl alcohol dehydrogenase* (SAD), *cinnamoyl‐CoA reductase* (CCR), *caffeic acid‐O‐methyltransferase*, and *flavonoid 3′‐O‐methyltransferase* that lead to lignin and flavonoid biosynthesis. In addition, the phenylpropanoid genes were co‐expressed with several *peroxidases*, suggesting they play an active role in regulating the phenylpropanoid pathway (Tables 2, S9a). In the arachidonic acid metabolism pathway, we found five genes, among them a gene similar to *gamma‐glutamyl transpeptidase 4*, which suggests a regulatory link between arachidonic acid metabolism and glutathione pathway (Tables 2, S9a). After 7 d of aphid feeding, the co‐expressed genes (modules ‘turquoise’ and ‘violet’) were enriched for amino sugar and nucleotide sugar metabolism and biosynthesis of nucleotide sugar (Fig. 5b). Among some of the main sugar genes are *hexokinase*, *UDP‐glucose dehydrogenase*, *mannose‐1‐phosphate guanyl transferase*, and *fructokinase*. These genes were co‐expressed with *chitinases*, including *chitinase 4*, *5*, and *8* (*pathogenesis‐related (PR)‐3*), suggesting combined inducible systemic defense mechanisms and nutritional reprogramming to orchestrate stress response (Tables 2, S9b). Finally, 21 d after aphid feeding (‘gray60’ module), we observed co‐expression among genes encoding for flavone and flavonol biosynthesis pathways, which is a branch of phenylpropanoid biosynthesis. These *UDP‐glucuronosyl/UDP‐glucuronosyltransferase* genes were co‐expressed with genes regulating biosynthesis of amino acid metabolic pathways, such as *phenylalanine* and *tyrosine*, that are substrates in the phenylpropanoid pathway (Fig. 5c; Tables 2, S9c,d).

**Table 2:** Enriched Kyoto Encyclopedia of Genes and Genomes (KEGG) pathways of the co‐expressed genes from selected modules in barley leaves upon rhizobacteria root colonization and shoot herbivory.

Time points	Module	KEGG pathway	Gene count	*P*‐value
24 h	Brown	Arachidonic acid metabolism	5	0.124
		Phenylpropanoid biosynthesis (lignin and flavonoids)	27	
		Plant‐hormone signal transduction (jasmonic acid, ethylene, auxin, cytokinin, MAPK)	33	
7 d	Turquoise and violet	Amino sugar and nucleotide sugar metabolism	37	**0.004**
		Biosynthesis of nucleotide sugars	23	**0.011**
21 d	Gray60	Flavone and flavonol biosynthesis	3	**< 0.001**
		Biosynthesis of amino acids (phenylalanine, tyrosine, and tryptophan biosynthesis)	2	**0.016**

Gene co‐expression analyses were performed on RNA‐seq data from barley (*Hordeum vulgare*) plants' roots colonized by rhizobacteria *Acidovorax radicis* or *Bacillus subtilis* and followed by infestation with the aphid *Sitobion avenae* at three different time points, including 24 h, 7 d, and 21 d after aphid feeding. *P*‐values indicate statistical significance with *P* < 0.05 indicated in bold. MAPK, mitogen‐activated protein kinase

### Hub genes analysis revealed that rhizobacteria induce defense and nutritional responses to suppress aphids on barley

**Fig. 6:**
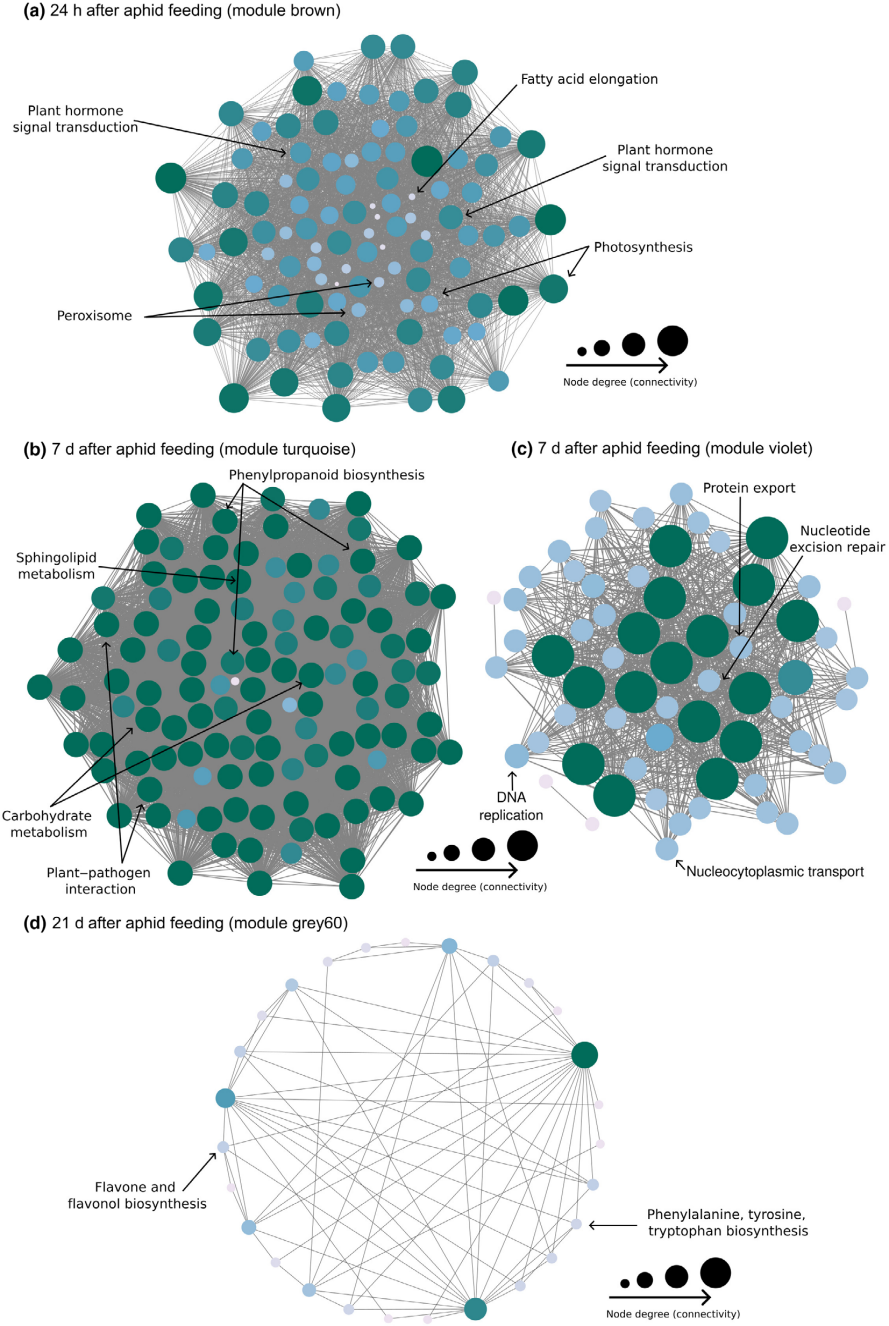
Network highlighting enriched Kyoto Encyclopedia of Genes and Genomes (KEGG) pathways from co‐expressed genes in barley leaves upon rhizobacteria root colonization and shoot herbivory. Weighted gene co‐expression network analysis (WGCNA) was used to identify key modules and hub genes associated with the treatment groups. The treatment groups were barley plants without treatment (*Control*), infested with the aphid *Sitobion avenae* (*Sa*), inoculated with the rhizobacteria *Acidovorax radicis* (*Ar*) or *Bacillus subtilis* (*Bs*), or inoculated with rhizobacteria and infested with aphid (*ArSa* or *BsSa*). In *Sa*, *ArSa* and *BsSa* plants, the aphid *S. avenae* was allowed to feed on plants for (a) 24 h (brown module), (b) 7 d (turquoise module), (c) 7 d (violet module), and (d) 21 d (gray60 module). Each node represents a gene, and the connecting lines (edges) between genes represent the co‐expression correlation. The node size and color are proportional to the degree of the input genes. The nodes in dark green suggest highly connected genes, while the lightly colored nodes represent less connected genes. The hub genes are labeled with the corresponding KEGG pathway term.

We constructed gene co‐expression networks based on weighting values and degree of genes in the selected modules of interest per time point (Fig. 6). In each selected module of interest per time point, we identified the hub genes, including 339 in module brown at 24 h, 906 in module turquoise and violet at 7 d, and 121 in module gray60 at 21 d (Table S10). KEGG enrichment analyses for each set of hub genes revealed consistent results with our previous enrichment analyses on differentially expressed genes and co‐expressed gene modules. After 24 h of aphid feeding, the hub genes in the module of interest (module brown, correlated with rhizobacteria inoculation effects) were enriched in the pathways: plant hormone signal transduction, fatty acid elongation, and MAPK signaling pathway. Interestingly, we found hub genes associated with photosynthesis, denoting plant growth (Fig. 6a; Tables 3, S11a). After 7 d of aphid feeding, only hub genes from the module turquoise (correlated with aphid feeding) were enriched, and this was in amino sugar and nucleotide sugar metabolism (Fig. 6b; Tables 3, S11b). After 21 d of aphid feeding, the hub genes in module gray60 (correlated with aphid feeding and *A. radicis* inoculation) were enriched in the pathways for flavone and flavonol biosynthesis, and phenylalanine, tyrosine, and tryptophan biosynthesis (Fig. 6c; Tables 3, S11c).

**Table 3:** Enriched Kyoto Encyclopedia of Genes and Genomes (KEGG) pathways of the hub genes from selected modules of co‐expressed genes in barley leaves upon rhizobacteria root colonization and shoot herbivory.

Time point	Module	Barley gene ID (HORVU.MOREX.r3.)	MM (*K*_ME_)	MM (*K*_ME_) *P* value	KEGG pathway	Gene count	*P* value
24 h	Brown	4HG0405320	0.84	5.10e‐07	Fatty acid elongation	1	0.003
		7HG0634340	0.96	9.63e‐13	Photosynthesis	4	0.011
		2HG0150570	0.92	4.08e‐10			
		2HG0141490	0.90	7.81e‐09			
		2HG0173200	0.88	2.99e‐08			
		3HG0239900	0.91	1.64e‐09	Plant hormone signal transduction	3	0.066
		4HG0380030	0.89	1.00e‐08	MAPK signaling pathway	2	
		1HG0024090	0.85	2.16e‐07	Peroxisome	2	0.157
7 d	Turquoise	4HG0379260	0.95	6.25e‐13	Plant–pathogen interaction	2	0.013
		5HG0473560	0.92	3.01e‐10			
		2HG0178020	0.96	4.21e‐13	Amino sugar and nucleotide sugar metabolism	2	0.014
		1HG0054950	0.93	2.49e‐11			
		5HG0512600	0.96	3.95e‐14	Phenylpropanoid biosynthesis	4	0.080
		2HG0112640	0.96	1.01e‐13			
		2HG0112630	0.94	2.02e‐11			
		3HG0312440	0.95	6.07e‐13	Sphingolipid metabolism	3	0.129
	Violet	5HG0471850	0.78	7.88e‐06	Nucleocytoplasmic transport	3	0.011
		1HG0080610	0.73	4.49e‐05	DNA replication/Base excision repair	2	0.016
		2HG0209100	0.70	0.000	Nucleotide excision repair/Basal transcription factors	2	0.031
		1HG0080610	0.73	4.49e‐05	Nonhomologous end‐joining	1	0.033
		5HG0504710	0.78	8.13e‐06	Protein export	1	0.141
21 d	Gray60	3HG0240580	0.90	4.18e‐09	Flavone and flavonol biosynthesis	3	< 0.001
		4HG0389730	0.95	1.04e‐11	Phenylalanine, tyrosine and tryptophan biosynthesis	1	0.043

Barley Gene ID (HORVU.MOREX.r3.), followed by the barley gene ID standard code; MM(*K*_ME_), modular membership connectivity value. Aphid: *Sitobion avenae*; Plant: barley *Hordeum vulgare*; Rhizobacteria: *Acidovorax radicis* and *Bacillus subtilis*. *P*‐values indicate statistical significance as extracted from analysis.

## Discussion

In this study, we explored global transcriptomic changes in barley plants inoculated with the rhizobacteria *A. radicis* or *B. subtilis*, correlated with suppression of aphid population growth on the plants. Rhizobacteria inoculation and aphids individually induced different plant responses; however, it was the combined and interactive effects that drove global gene expression. After only 24 h of aphid feeding, inoculated plants responded differently to aphids than uninoculated plants, particularly related to responses in the phenylpropanoid, glutathione, and phytohormone pathways. As aphids began to reproduce, by day 7 we observed stronger aphid‐induced plant responses related to the induction of the phenylpropanoid pathway, but also nutrient pathways for carbohydrates, amino acids, and sugars. By day 21, aphid colonies were well established, and we observed further changes in plant defense pathways, including flavones and flavonoids, as well as genes related to plant responses to tissue damage and repair. Although the two rhizobacteria are distinct species with unique traits, their shared functional similarities in shaping plant response to aphid infestation suggest shared mechanisms that prime plants for aphid‐induced stress. This convergence might reflect broad‐spectrum plant responses triggered by beneficial rhizobacteria (Bruto *et al*., 2014; Rosier *et al*., 2018), emphasizing their potential to enhance plant resilience to herbivores. Our study design did not use sterile soil, but rather low‐microbial diversity potting substrate (e.g. Zytynska *et al*., 2020); thus, we do not assume that the single inoculated strain directly drives all these plant responses. It is feasible, and likely, that the inoculated strain can shift the functioning of the resident microbiome, potentially recruiting additional rhizobacteria as ‘supportive strains’ to achieve the strong outcomes (Castro‐Sowinski *et al*., 2007; Hu *et al*., 2021), and benefiting persistence of the inoculated strain (Eckshtain‐Levi *et al*., 2020). Nevertheless, our robust experimental design allows us to compare the results of microbial inoculation to controls and across strains.

During the early stages of aphid feeding, many of the defensive genes were suppressed on inoculated plants compared to uninoculated plants. It is possible that rhizobacteria may dampen and fine‐tune plant defense responses to early aphid feeding when only a few aphids are present, thus conserving or reallocating energy for plant growth. This may explain our current results and previous work on this system showing that plant growth promotion was strongest in the early growth stages of the plant (Zytynska *et al*., 2020; Xi *et al*., 2024), and also how diverse strains of rhizobacteria can simultaneously promote growth and induce resistance to herbivores (Rashid *et al*., 2017). We further identified hub genes encoding for photosynthesis during early aphid feeding, supporting that rhizobacteria inoculation led to improved plant growth. Induction of defense and nutritional pathways at the 7‐d time point, with weaker impacts of rhizobacterial inoculation, suggests that rhizobacteria‐induced responses are important to establish as early as possible during aphid colonization. Inoculation of broad bean plants with *Bacillus amyloliquefaciens* altered aphid feeding behaviour, with increased nonprobing and salivation events (Serteyn *et al*., 2020), suggesting that aphids may take longer to settle on inoculated plants, and thus longer to initiate reproduction. A combination of behavioral changes and impacts of plant defenses on aphid fitness, resulting in reduced offspring production, are key to reducing aphid populations (Smith & Chuang, 2014). Similarly, any nutritional changes in the plants may lead to imbalances that can impact aphid fitness, but we cannot rule out that the aphids themselves can manipulate the environment to counter plant defensive mechanisms (Züst & Agrawal, 2016). Notably, aphid infestation results in plant part‐specific changes in carbon–nitrogen allocation and phloem–sap composition, highlighting their ability to construct their own niche and optimize food quality (Girousse *et al*., 2005; Jakobs *et al*., 2019). These changes in nutrients might further support activation of signaling pathways and production of defense compounds, as well as boosting plant growth and development (Sun *et al*., 2020).

We found that both the rhizobacteria *A. radicis* and *B. subtilis* altered the expression of genes in the phenylpropanoid biosynthesis pathway. The phenylpropanoid metabolism pathway codes for many different secondary metabolites and leads to the production of lignin and flavonoids (Vogt, 2010). Phenylpropanoid defensive genes are known to be induced by herbivore feeding, and expression varies among resistant and susceptible plant varieties (Ramaroson *et al*., 2022). For example, resistance to aphids in rose plants was linked to the biosynthesis of secondary metabolites, including phenylpropanoids, alkaloids, and flavonoids (Dong *et al*., 2024). Previous work on *A. radicis* also observed that this rhizobacteria induced the phenylpropanoid and flavonoid pathway in barley linked to microbial N‐acyl‐homoserine lactone (AHL) production (Han *et al*., 2016). AHLs have been linked to increased plant resilience to stress with correlated changes in root architecture, as well as the plant transcriptome and metabolome (Babenko *et al*., 2022). However, the *B. subtilis* strain does not synthesize AHL molecules (Blake *et al*., 2021), and thus, while the shoot expression profiles may be similar between the two rhizobacteria, the mechanisms of effect likely differ. *Bacillus subtilis* interacts with plant roots in various ways, including secreting enzymes and phytohormones that trigger systemic resistance in the plant by activating JA and SA signaling pathways (Blake *et al*., 2021). We also observed rhizobacteria induction of plant phytohormones, including jasmonyl‐*l*‐isoleucine synthase encoding for JA signaling and several ET receptors and transcription factors, confirming these pathways of interest in our system.

A novel result in our study was that rhizobacteria activated arachidonic acid metabolism, which has previously been linked to plant defense against phytopathogens (Dedyukhina *et al*., 2014). We observed very low levels of expression of these genes, but with potential for biological relevance as high arachidonic acid concentrations can induce necrosis and accumulation of phytoalexins, while low amounts can elicit systemic and prolonged resistance to phytopathogens (Ivanyuk *et al*., 1990; Ozeretskovskaya, 1994; Dedyukhina *et al*., 2014). Here, we speculate that this contributed to our observed microbial‐induced resistance against aphids, and thus requires further study.

Rhizobacteria inoculation of plant roots can thus alter plant defenses directly, as shown by the various pathways that are up‐ and downregulated in inoculated plants compared to controls in our study. Microbial inoculation can also prime plants, where plant defenses only activate when triggered by insect feeding (Conrath, 2011; Kim & Felton, 2013). When comparing gene expression of plants inoculated with rhizobacteria without aphids and inoculated plants with aphids, we can begin to disentangle induced and primed responses in the plants. For *A. radicis*, the suppression of phenylpropanoid‐related genes occurred independently of aphid feeding, while the induction of glutathione was a primed response activated only on aphid‐present plants in the later time point when aphid density was high. A study on *Arabidopsis* similarly concluded that the maintenance of glutathione levels played a major role in priming and defending against plant stress (Csiszár *et al*., 2018). For the phenylpropanoid pathway, the increased expression of genes in this pathway in the later time points could be attributed to aphid induction, but the strength of expression varied with rhizobacteria inoculation for both *A. radicis* and *B. subtilis*. This is likely a combination of priming but also a response to the variable aphid densities due to earlier suppression effects. While the population density of herbivores impacts their physiological response, for example, through crowding effects (Applebaum & Heifetz, 1999), this can also alter their responses to induced plant defenses (Frost *et al*., 2008; Züst & Agrawal, 2016). Further experimental work needs to disentangle the effects of insect suppression that result from microbial‐induced plant defenses and the consequences of this reduced aphid density on long‐term colony responses to microbial inoculation.

In conclusion, the rhizobacteria *A. radicis* and *B. subtilis* can suppress aphid populations on barley by modulating and priming a set of plant defensive pathways. These include defense pathways such as phenylpropanoid, glutathione, and phytohormones, as well as several nutritional pathways. Our data suggest that microbial inoculation of barley against aphids is a dynamic phenomenon that acts through various pathways that prime and induce plant resistance (defenses) and tolerance (nutrition and growth) to the growing aphid population. Moreover, we speculate that the strength of these plant defenses is modulated by the rhizobacteria in response to aphid density or population expansion. Aphid populations in crop fields tend to be dispersed across multiple host plants with lower numbers per host, unless in the event of a local outbreak when numbers dramatically increase. Therefore, if microbial inoculants can benefit the plant by enabling higher energy allocation to growth for plants when only none or a few aphids are feeding, but enable a quick defensive response to increasing aphid densities, this could provide efficient crop protection.

## Competing interests

None declared.

## Author contributions

SEZ conceptualized the project idea and obtained grant funding. CMM designed the experiments. SEZ and CMM conducted the experiments, analyzed the experimental data, and interpreted the results. CMM was involved in processing samples, including RNA extraction, transcriptomics data analysis, and results interpretation. SEZ and CMM were involved in manuscript writing.

## Disclaimer

The New Phytologist Foundation remains neutral with regard to jurisdictional claims in maps and in any institutional affiliations.

## Supporting information


**Fig. S1** Gene co‐expression network analyses in barley leaves upon rhizobacteria root colonization and shoot herbivory.
**Fig. S2** Average shoot lengths of barley var. Irina with and without rhizobacteria inoculation and shoot herbivory, across time points.
**Fig. S3** Average root lengths of barley var. Irina with and without rhizobacteria inoculation and shoot herbivory, across time points.

**Table S1** Overview of raw, trimmed, and aligned reads.
**Table S2** Summary of differentially expressed genes in barley leaves upon rhizobacteria root colonization and shoot herbivory.

**Table S3** Summary of the number of genes per enriched KEGG pathways for each treatment and time point.

**Table S4** Enriched KEGG pathways for each treatment group after 24 h of aphid feeding.

**Table S5** Enriched KEGG pathways for each treatment group after 7 d of aphid feeding.

**Table S6** Enriched KEGG pathways for each treatment group after 21 d of aphid feeding.
**Table S7** Modules of co‐expressed genes and number of genes per module in barley leaves upon rhizobacteria root colonization and shoot herbivory.

**Table S8** Summary statistics of the module trait (treatment group) relationship per time point, including after 24 h, 7 d, and 21 d of aphid feeding.

**Table S9** Enriched KEGG pathways for selected module of interest per time point, including after 24 h, 7 d, and 21 d of aphid feeding.

**Table S10** The number of hub genes extracted in each selected module of interest per time point, after 24 h, 7 d, and 21 d of aphid feeding.

**Table S11** Enriched KEGG pathways for hub genes in the selected module of interest per time point, including after 24 h, 7 d, and 21 d of aphid feeding.
Please note: Wiley is not responsible for the content or functionality of any Supporting Information supplied by the authors. Any queries (other than missing material) should be directed to the *New Phytologist* Central Office.

## Data Availability

Raw sequencing data are available from the European Nucleotide Archive (ENA; https://www.ebi.ac.uk/ena) under the study PRJEB90049 and accession no. ERS24808620‐89.
