# Supplementary material for: Rhizobacteria prime the activation of plant defense and nutritional responses to suppress aphid populations on barley over time
Source: New Phytol. 2025 Jun 26;247(5):2390–405. doi: 10.1111/nph.70319 (PMC12329176; doi:10.1111/nph.70319)
Supplement: Supplementary file 1 — Fig. S1 Gene co‐expression network analyses in barley leaves upon rhizobacteria root colonization and shoot herbivory. Fig. S2 Average shoot lengths of barley var. Irina with and without rhizobacteria inoculation and shoot herbivory, across time points. Fig. S3 Average root lengths of barley var. Irina with and without rhizobacteria inoculation and shoot herbivory, across time points. [file NPH-247-2390-s007.pdf]

New Phytologist **Supporting Information**

Article title: **Rhizobacteria prime the activation of defence and nutritional responses to suppresses aphid populations on barley**

Authors: Crispus M. Mbaluto and Sharon E. Zytynska

Article acceptance date: 04 June 2025

The following Supporting Information is available for this article:

Fig. S1 Gene co-expression network analyses in barley leaves upon rhizobacteria root colonization and shoot herbivory

Fig. S2 Average shoot lengths of barley var. Irina with and without rhizobacteria inoculation (Bs or Ar) and shoot herbivory (Sa) across time points**.**

Fig. S3 Average root lengths of barley var. Irina with and without rhizobacteria inoculation and shoot herbivory, over time.

Table S2 Summary of differentially expressed genes in barley leaves upon rhizobacteria root colonization and shoot herbivory.

Table S7 Modules of co-expressed genes and number of genes per module in barley leaves upon rhizobacteria root colonization and shoot herbivory

Supplied as separate .xlsx files:

Table S1 Overview of raw, trimmed and aligned reads

Table S3 Summary of number of genes per enriched KEGG pathways for each treatment and timepoint.

Table S4 Enriched KEGG pathways for each treatment group after 24 hours of aphid feeding

Table S5 Enriched KEGG pathways for each treatment group after 7 days of aphid feeding

Table S6 Enriched KEGG pathways for each treatment group after 21 days of aphid feeding

Table S8 Summary statistic of the module trait (treatment group) relationship per timepoint, including after 24 hours, 7 and 21 days of aphid feeding

Table S9 Enriched KEGG pathways for selected module of interest per timepoint, including after 24 hours, 7 and 21 days of aphid feeding

Table S10 The number of hub gene extracted in each selected module of interest per timepoint, after 24 hours, 7 and 21 days of aphid feeding

Table S11 Enriched KEGG pathways for hub genes in the selected module of interest per timepoint, including after 24 hours, 7 and 21 days of aphid feeding

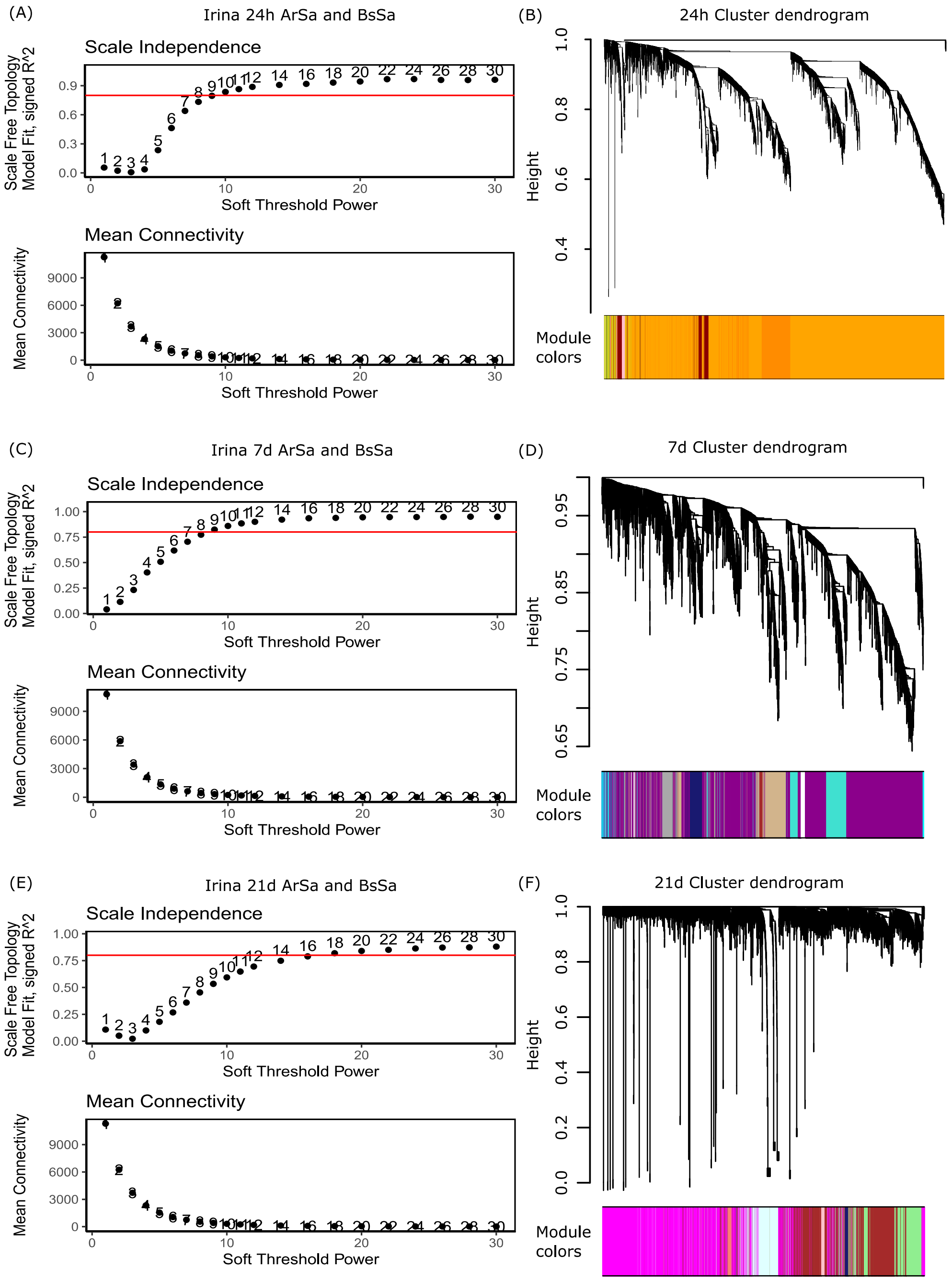

Fig. S1 Gene co-expression network analyses in barley leaves upon rhizobacteria root colonization and shoot herbivory. Weighted gene co-expression analyses was used to determine gene modules soft thresholds in panels **a,c,e** and the co-expressed gene module by the dynamic tree cut and then merged based on similarity in expression pattern in panel **b,d,f**. Colors along the bottom indicate module assignment. Grey bars signify genes that were not assigned to any module.

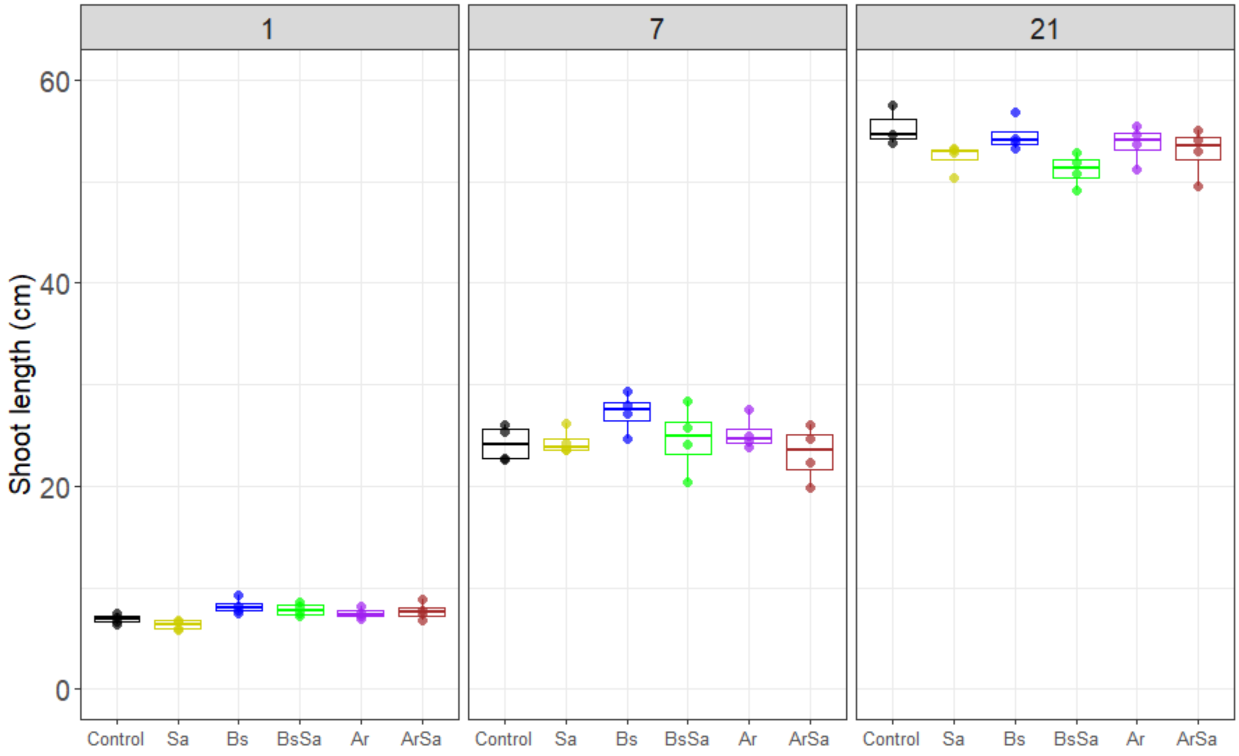

Fig. S2 Average shoot lengths of barley var. Irina with and without rhizobacteria inoculation (Bs or Ar) and shoot herbivory (Sa), on plants across time points. Including uninoculated (control) plants and inoculated plants with rhizobacteria *Bacillus subtilis* (Bs) or *Acidovorax radicis* (Ar), and infested with aphid *Sitobion avenae* (Sa). Rhizobacteria inoculated with aphids denoted as BsSa or ArSa. Plants harvested at 24 hours (1), 7 days (7) and 21 days (21). Boxplots show the median (horizontal line), interquartile range (box), and interquartile range (whiskers). Points represent individual data values.

Bacteria main effect

day 1: $F_{2,20}$=9.51, P=0.001, day7: $F_{2,20}$=1.58, P=0.231, day 21: $F_{2,19}$=0.37, P=0.698.

Aphid main effect

day 1: $F_{1,20}$=0.52, P=0.481, day7: $F_{1,20}$=2.62, P=0.121, day 21: $F_{1,19}$=9.39, P=0.006.

No significant interactions

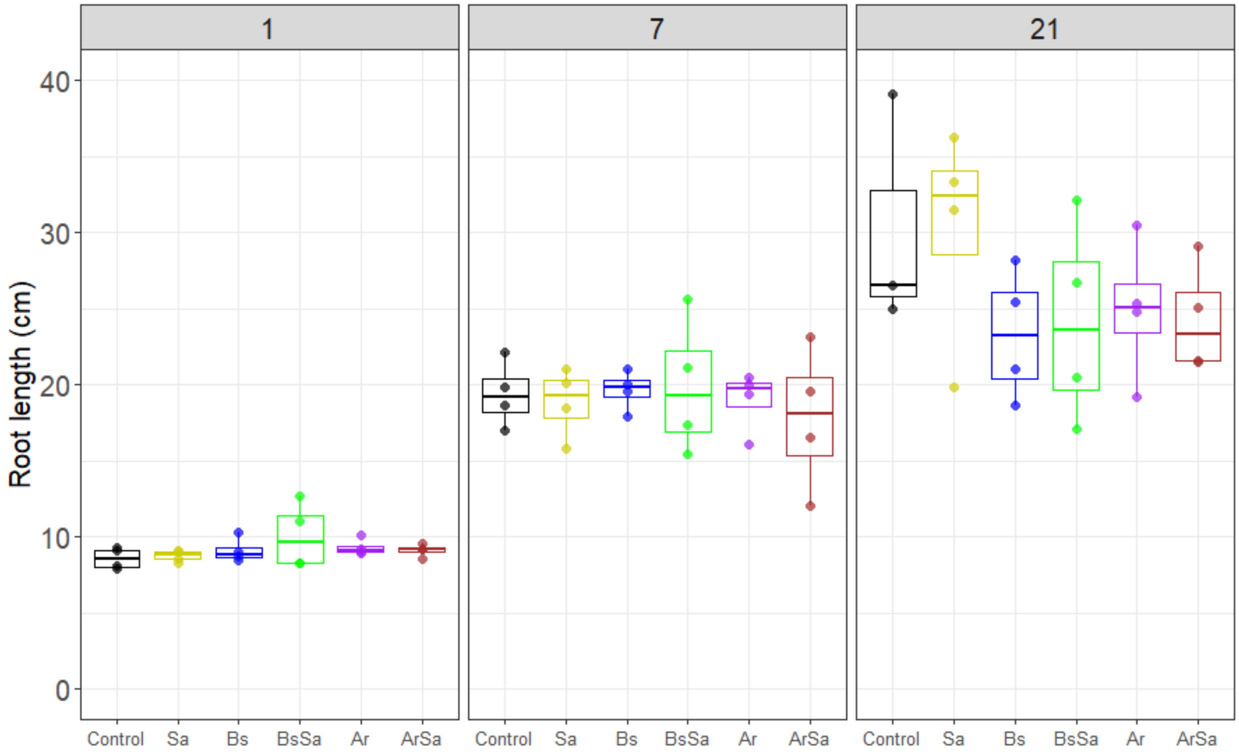

Fig. S3 Average root lengths of barley var. Irina with and without rhizobacteria inoculation and shoot herbivory, over time. Including uninoculated (control) plants and inoculated plants with rhizobacteria *Bacillus subtilis* (Bs) or *Acidovorax radicis* (Ar) and infested with aphid *Sitobion avenae* (Sa). Plants harvested at 24 hours (1), 7 days (7) and 21 days (21). Boxplots show the median (horizontal line), interquartile range (box), and interquartile range (whiskers). Points represent individual data values.

Bacteria main effect

day 1: $F_{2,20}$=1.68, P=0.212, day7: $F_{2,20}$=0.42, P=0.665, day 21: $F_{2,19}$=3.02, P=0.073.

Aphid main effect

day 1: $F_{1,20}$=0.58, P=0.454, day7: $F_{1,20}$=0.17, P=0.689, day 21: $F_{1,19}$=0.01, P=0.979.

No significant interactions

Table S2 Summary of differentially expressed genes in barley leaves upon rhizobacteria root colonization and shoot herbivory, the aphid S. avenae was allowed to feed on plants for 24 hours, 7 and 21 days. No-herbivory plants harvested at the same time point.

**Treatment**	**Controls**	**Time points**								
		**DEGs at 24 hours**			**DEGs at 7 days**			**DEGs at 21 days**		
		Total	Up	Down	Total	Up	Down	Total	Up	Down
Sa	vs. Control	331	212	119	656	540	116	1868	1432	436
Ar		1416	54	1362	449	104	345	1428	1148	280
Bs		927	413	514	417	83	334	1533	977	556
ArSa		1242	114	1128	871	701	170	1890	1622	268
BsSa		1463	136	1327	793	617	176	1694	1332	362
ArSa	vs. Ar	233	196	37	893	760	133	587	435	152
BsSa	vs. Bs	335	96	239	812	684	128	739	577	162
ArSa	vs. Sa	1420	180	1240	226	111	115	558	422	136
BsSa		1495	126	1369	244	115	129	555	351	204

Notes: Uninoculated (control) plants and inoculated plants with rhizobacteria *Bacillus subtilis* (Bs) or *Acidovorax radicis* (Ar), and infested with aphid *Sitobion avenae* (Sa). Rhizobacteria inoculated with aphids denoted as BsSa or ArSa. Samples were collected at end of each feeding duration, sequenced and DE genes identified using R package limma

Table S7 Modules of co-expressed genes and number of genes per module in barley leaves upon rhizobacteria root colonization and shoot herbivory. The module 14-grey signifies genes that were not assigned to any module. In bold are the modules and genes selected as the modules of interest

**Module color**		**Time point**		
		**24 hours**	**7 days**	**21 days**
1	Black	660	909	615
2	Blue	3068	3235	1980
**3**	**Brown**	**2555**	2207	1293
4	Cyan	196	314	180
5	Darkgreen	92	206	31
6	Darkgrey	65	182	-
7	Darkmagenta	-	58	-
8	Darkolivegreen	-	76	-
9	Darkorange	-	121	-
10	Darkred	109	206	40
11	Darkturquoise	69	204	-
12	Green	2205	1064	1145
13	Greenyellow	359	340	412
14	Grey	2685	2320	9750
**15**	**Grey60**	148	246	**85**
16	Lightcyan	154	290	92
17	Lightgreen	136	245	79
18	Lightyellow	136	245	65
19	Magenta	426	507	506
20	Midnightblue	174	299	147
21	Orange	55	135	-
22	Paleturquoise	-	97	-
23	Pink	496	548	522
24	Purple	382	402	438
25	Red	894	931	907
26	Royalblue	123	238	60
27	Saddlebrown	-	107	-
28	Salmon	209	329	247
29	Sienna3	-	50	-
30	Skyblue	-	110	-
31	Skyblue3	-	35	-
32	Steelblue	-	99	-
33	Tan	224	332	285
**34**	**Turquoise**	4451	**3353**	2328
**35**	**Violet**	-	**90**	-
36	White	-	112	-
37	Yellow	2214	1263	1250
38	Yellowgreen	-	43	-
